# Why the Shaw–Mohler equation works and when it doesn't

**DOI:** 10.1098/rsbl.2023.0499

**Published:** 2024-02-28

**Authors:** Martin Burd

**Affiliations:** Department of Biology, Indiana University Herbarium, Indiana University, Bloomington, IN, USA

**Keywords:** hermaphrodite, reproductive ecology, sex allocation, sex ratio

## Abstract

Fitness gain curves were introduced into the framework of the Shaw–Mohler equation, the foundation of sex allocation theory. I return to the Shaw–Mohler equation to consider how it embodies the rare-sex advantage underlying frequency-dependent selection on the sex ratio. The Shaw–Mohler formulation is based on the numbers of males and females randomly mating in a panmictic population. Gain curves are meant to describe reproductive success through male and female functions in hermaphrodites and have been inserted in place of male and female numbers in the Shaw–Mohler equation. In doing so, gain curves bypass consideration of the implicit mating process in the Shaw–Mohler argument and can lead to anomalies like unequal total male and female fitness in a population. If gain curves truly represent fitness gain, equality of total male and female fitness requires a constant sex allocation of equal resource investment into male and female functions. The blurring of input with fitness outcome has led to misinterpretation of what gain curves mean in reproductive ecology. They can describe a particular reproductive ecology, such as diminishing fitness returns on resource investment, but lack causal efficacy with respect to sex allocation.

## 1. Introduction

Shaw & Mohler [1] formulated a model of optimal sex ratios using an argument later recognized to be an early form of an evolutionarily stable strategy (ESS) analysis [2]. The eponymous equation from their model has become a foundation of sex allocation theory. But in the course of use it has been modified, and it is worth returning to the source to consider how the Shaw–Mohler equation functions, how it represents the frequency-dependent selection that governs sex allocation [3], and whether it still works with its modifications. In particular, I wish to consider whether and how the Shaw–Mohler equation accommodates fitness gain curves, which Charnov [2,4] introduced into the Shaw–Mohler framework to explain sexual resource allocation in hermaphroditic organisms. Gain curves have become a staple tool for theoretical modelling [5–12] and for interpreting empirical data on reproductive success in hermaphroditic plants and animals [13–21]. The issue is therefore of considerable importance.

## 2. The Shaw–Mohler equation

I will adopt the notation of Shaw & Mohler [1] with one exception: they labelled the genetic contribution to subsequent generations of the hypothetical mutant in their argument as *C*_m_. I instead denote mutant fitness as *w*.

**Figure 1.**
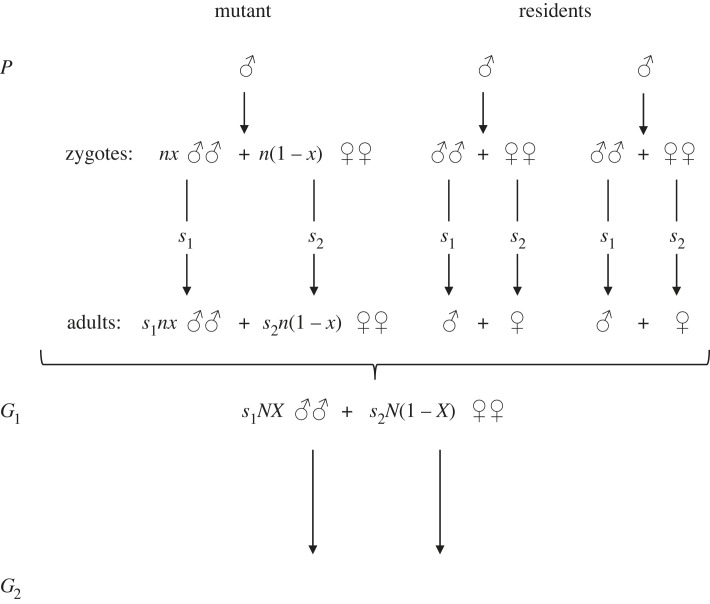
The Shaw–Mohler formulation of reproductive success across three generations (adapted with permission from Shaw & Mohler [1, fig. 1]).

Figure 1 depicts the multigenerational scheme of their analysis. Males of the parental generation collectively sire *NX* male offspring and *N*(1 – *X*) female offspring, and with sex-specific survival rates *s*_1_ and *s*_2_ the adult population of generation G_1_ comprises *s*_1_*NX* males and *s*_2_*N*(1– *X*) females. Shaw & Mohler [1] initially omitted the survival rates and then added them to show that they do not affect the optimal sex ratio; figure 1 simply includes the survival terms from the start. In current terminology, *X* would be labelled the resident sex allocation.

Within the parental generation is a focal male, now termed the mutant, that sires *nx* male offspring and *n*(1 – *x*) female offspring in G_1_. That is, the mutant employs sex ratio strategy *x* rather than *X*. Shaw & Mohler [1] determine the mutant's genetic contribution to the grand-offspring generation, G_2_, via the reproductive fate of its G_1_ cohort. The reasoning is straightforward. All G_1_ males collectively contribute half the (nuclear) genes that are transmitted to G_2_, and so the mean genetic contribution per male among the *s*_1_*NX* adult males in G_1_ must be 1/(2*s*_1_*NX*). The male offspring of the mutant male parent will therefore contribute, on average, *s*_1_*nx*/(2*s*_1_*NX*) of the genes transmitted to G_2_. Had we followed a focal female in the parental generation, the same reasoning would lead to a contribution of *s*_2_*n*(1 – *x*)/[2*s*_2_*N*(1 – *X*)] from its G_1_ female offspring to G_2_. The survival rates *s*_1_ and *s*_2_ appear in both the numerator and denominator of the male and female fitness expressions and therefore resolve to a factor of unity, whence the conclusion that post-investment survival of offspring, even if different between the sexes, is irrelevant to sex allocation. A further implication is that sexual differences in survival might leave different numbers of adult males and females in the mating pool.

Since the genetic contribution of a focal individual is halved with every successive generation, the contribution of the mutant in P to G_2_ through both its male and female offspring is

2.1 $$w(x,X)=\frac{1}{4}\left(\frac{nx}{NX}+\frac{n(1-x)}{N(1-X)}\right)=\frac{1}{4}\left(\frac{m}{M}+\frac{\;f}{F}\right),$$

in which *m* and *f* are the numbers of male and female zygotes produced by the focal mutant and *M* and *F* are the total numbers of male and female zygotes in the G_1_ generation. The motif *m*/*M* + *f*/*F* or some variation is the core of what is referred to as the Shaw & Mohler equation.

Several features of this scheme deserve comment. Figure 1 does not specify the number of adults in generation P nor the number of zygotes or adults in G_2_, only the number of males and females in the G_1_ mating arena. Since the zygotes of G_2_ (or adults, depending on which we are counting—as noted above, survival can be ignored) represent the total fitness of the G_1_ generation, the Shaw–Mohler equation does not specify the fitness outcome of the mating interactions that take place among the G_1_ males and females. It suffices that the mutant receive its fair share of the unstated total fitness based on the proportion of its sons and daughters in the G_1_ mating pool, embodied in the ratios *m*/*M* and *f*/*F*. Of course, the implicit fitness can be made explicit. If G_2_ comprises *Z* total zygotes, the Shaw–Mohler equation becomes

2.2 $$w(x,X)=\frac{1}{4}\left(\frac{m}{M}+\frac{\;f}{F}\right)Z.$$

If one sex is more numerous (i.e. *M* ≠ *F*), perhaps due to sex-specific survival, each individual of that sex will acquire, on average, a smaller share of the total fitness *Z*. In this way, the Shaw–Mohler equation adjusts the reproductive value of an individual of each sex relative to the number of competitors of that sex, and so implicitly creates the frequency-dependent selection at the heart of sex ratio evolution [3,22,23].

The fitness of the mutant is a function of both its own sex allocation, *x*, and the common sex allocation of the residents, *X*. Shaw & Mohler [1] depict this dual dependence in a figure, from which they infer that the evolutionarily stable allocation occurs when *x* = *X* = ½. Current practice in an ESS analysis reaches the same end in a more general fashion. The optimal sex allocation is the value *x* = *X* = X* that satisfies the condition ∂*w*/∂*x*|_*x* = *X*_ = 0. For equation (2.1), this condition implies that *X* = 1 – *X*, which has the solution X* = ½. An additional condition to exclude minima and inflection points must be met to ensure that this point represents an ESS [24] and it is straightforward to confirm that this condition is satisfied.

## 3. Use of relative terms in the Shaw–Mohler equation

The *m*/*M* + *f*/*F* core of equation (2.1) might be modified to $m/\bar{m}+f/\bar{\;f}$, in which the denominators represent not the total numbers of males and females in G_1_ but the average number of sons and daughters produced by a single parent of generation P. That is, mutant fitness is expressed relative to mean individual fitness in the population. If the average parent with a resident sex allocation strategy contributes either sperm or eggs to *n* offspring, as the mutant does, then equation (2.2) becomes

3.1 $$w(x,X)=\frac{1}{4}\left(\frac{m}{\bar{m}}+\frac{\;f}{\bar{\;f}}\right)\bar{Z}=\frac{1}{4}\left(\frac{nx}{nX}+\frac{n(1-x)}{n(1-X)}\right)\bar{Z}.$$

The ratios $m/\bar{m}+f/\bar{\;f}$ are valid expressions of the mutant's fair share of mean fitness $\bar{Z}$ because the average production of sons and daughters per resident parent bears a simple relationship to the total number of males and females in G_1_, and panmictic mating and the ‘fair share’ principle for the mutant are unchanged.

Since we count fitness by the production of offspring, we might write equation (3.1) not in terms of numbers of sons and daughters but in terms of fitness derived through male and female pathways of fitness accrual:

3.2 $$w(x,X)=\frac{1}{4}\left(\frac{w_{m}}{{\bar{w}}_{m}}+\frac{w_{f}}{{\bar{w}}_{f}}\right),$$

in which *w*_m_ and *w*_f_ represent the mutant's sex-specific fitness and ${\bar{w}}_{m}$ and ${\bar{w}}_{f}$ are the corresponding mean individual fitnesses for the resident type. This version of the Shaw–Mohler equation is the form in which it is often presented (e.g. [22,23,25]).

A problem with equation (3.2) is that the fitness terms are remote from the mating process implied by equation (2.1) and from the total fitness explicit in equation (2.2), so there is no indication of how the terms of equation (3.2) arise. Of course, the assumptions underlying the terms can be made explicit [23]. Yet a problem remains. A correctly formed Shaw–Mohler equation (2.1) based on numbers of competing males and females will be compatible with the fitness version in equation (3.2), but the converse is not necessarily true: we cannot necessarily use any fitness functions in equation (3.2) and produce a valid Shaw–Mohler formulation of a mutant's fair share of total fitness *Z*. The fitness functions must be tested on their own merits for biological legitimacy.

## 4. Gain curves

With hermaphrodites we are no longer dealing with diploid male and female zygotes or adults, since all parents and offspring are hermaphrodites. But male and female gametes (or entities that eventually produce gametes, such as microspores and megaspores or pollen and ovules) can be interposed into the Shaw–Mohler generational scheme, so that the terms *x* and *X* designate not a sex ratio but the sexual division of a resource pool in which *x* represents the fraction given to sperm production and 1 – *x* to eggs. However, size differences between the male and female entities (and we can include the *pro rata* ancillary costs of dispersing them or otherwise enabling their mating potential) no longer allow *a priori* specification of the total numbers produced by the mutant or by the population; e.g. we cannot suppose that a mutant produces *n* gametes of which *nx* are male and *n*(1 – *x*) are female because the total number *n* is itself a function of *x*. Suppose, however, that a mutant hermaphrodite can produce *m*(*x*) sperm and *f*(*x*) eggs, while a parental population of *H*_P_ hermaphrodites collectively produces *H*_P_*m*(*X*) sperm and *H*_P_*f*(*X*) eggs. This leads to a Shaw–Mohler equation in conventional form for the gamete rather than zygote stage of the life cycle:

4.1 $$w(x,X)=\frac{1}{2}\left(\frac{m(x)}{H_{P}m(X)}+\frac{\;f(1-x)}{H_{P}f(1-X)}\right)\propto \;\left(\frac{m(x)}{m(X)}+\frac{\;f(1-x)}{\;f(1-X)}\right).$$

We need only define functions *m*(*x*) and *f*(*x*) that relate resource investment to the number of gametes produced.

Charnov [2,4] proposed that these functions be *m*(*x*) = *x^b^* and *f*(*x*) = 1 – *x*, which he termed *gain curves*. (He used the notation *r^n^* and 1 – *r*, but to preserve the notation of Shaw & Mohler [1], in which *n* has another meaning, I use *x^b^* and 1 – *x*.) Charnov [2, p. 220] conceived of these functions not in terms of the numbers of male and female actors in equation (2.1) but in terms of male and female fitness in equation (3.2). Arguing from Bateman's principle [26], he suggested that *b* would typically be less than unity, so that the ability of an adult hermaphrodite to obtain fertilizations through sperm production would wane as more and more sperm were produced, while its success through egg production would be proportional to resource investment. Equation (4.1) with these gain curves yields

4.2 $$w(x,X)=\frac{1}{2}\left(\frac{x^{b}}{H_{P}X^{b}}+\frac{1-x}{H_{P}(1-X)}\right),$$

which leads to an ESS allocation of X* = *b*/(1 + *b*) [2].

The earlier caution about the implicit context from which the fitness terms of equation (3.2) gain their meaning now comes into play. If we insist that *x^b^*, *X^b^*, (1 – *x*), and (1 – *X*) represent fitness itself (rather than entering into ratios that express a fair share of total fitness), then, total male fitness under a universal allocation X* must be *H*_P_(X*)*^b^*, and similarly total female fitness must be *H*_P_(1 – X*). Because these two must be equal, (X*)*^b^* must equal (1 – X*). If we substitute the putative ESS X* = *b*/(1 + *b*) we obtain

$${\left(\frac{b}{1+b}\right)}^{b}=1-\frac{b}{1+b}=\frac{1}{1+b},$$

which has a solution only at *b* = 1, and therefore an allocation of X* = ½. Any gain curve exponent other than *b* = 1 violates a fundamental requirement of sexual reproduction.

The relative fitness form of the Shaw–Mohler equation (3.2) will approve of gain curves that we know are biologically false. Suppose that male fitness is 100 times greater than female fitness, whatever it is. If the female curve is *f*(*x*) = 1 – *x*, the male curve is *m*(*x*) = 100·(1 – *x*). From equation (3.2) (which considers two generations of transmission and so a coefficient of ¼), the mutant's relative male and female fitness would be

4.3*a* $$w_{m}(x,X)=\frac{1}{4}\cdot \frac{100(1-x)}{100(1-X)},$$

4.3*b* $$w_{f}(x,X)=\frac{1}{4}\cdot \frac{\left(1-x\right)}{\left(1-X\right)},$$

and at a universal allocation *x* = *X* = X* these relative fitnesses would be equal. Yet we have used gain curves that are biologically impossible by design. Thus, equation (3.2) can be legitimate, but it is not universally legitimate; the gain curves underlying fitness must satisfy biological requirements.

We need not dispense with gain curves representing absolute fitness, but to fold them into the Shaw–Mohler framework, total population fitness *Z* (and not just male fitness) must be made a function of a gain curve (because there is no male fitness acquisition without equivalent female fitness in a sexually reproducing population). A simple way to do this is to recognize that a given resource investment allows production of a given number of eggs, and total egg production across all hermaphrodites in the population sets the maximum number of zygotes that might be formed. Let a unit of resource allocation yield *k*_m_ male gametes or *k*_f_ female gametes. Total egg production in the population would then be *H*_P_*k*_f_(1 – *X*). These eggs become zygotes only if fertilized, so to represent the diminishing marginal effectiveness of male investment in this task, total egg number can be multiplied by *X^b^* (*b* < 1), which will always take a value from 0 to 1*.* Some eggs remain unfertilized if *X* < 1. As *X* approaches 1, egg fertilization approaches complete success but fewer resources are available to produce eggs. Equation (4.1) with this explicit definition of *Z* becomes

4.4 $$w(x,X)=\;\frac{1}{2}\left[\frac{k_{m}x}{H_{P}k_{m}X}+\frac{k_{f}(1-x)}{H_{P}k_{f}(1-X)}\right]H_{P}k_{f}(1-X)X^{b}.$$

The derivative of (4.4) is

4.5 $$\frac{dw}{dx}=\left[\frac{1}{H_{P}X}-\frac{1}{H_{P}(1-X)}\right]H_{P}k_{m}(1-X)X^{b},$$

and the ESS requirement of d*w*/d*x* = 0 is satisfied only at X* = ½. The reason is easily grasped. For nonlinear gain curves to represent male fitness they must affect *Z*, but *Z* has equal consequences for male and for female fitness, and so the nonlinearity has no special implication for sex allocation. Total population fitness is affected by *b*, but not optimal sex allocation.

If we allow *m*(*x*) = *x^b^* and *f*(*x*) = 1 – *x* to refer to sperm and egg production rather than the fitness outcome of a mating process, then X* = *b*/(1 + *b*) is an acceptable ESS, even for *b* ≠ 1, but we have abandoned the fitness meaning given to gain curves at their inception and ever since. We must also find new justifications for the forms *x^b^* and 1 – *x* as gamete production functions. A common argument [2] is that fixed start-up costs for reproduction (such as the pedicel, receptacle, sepals, and petals of flowers in angiosperms) allow hermaphrodites to produce more gametes than single-sex individuals because the fixed costs can be shared by both sex functions. It is not immediately obvious, however, that this sharing argument privileges sperm production with the advantage implied by *x^b^* and *b* < 1. Functions *m* = *x* and *f* = (1 – *x*)*^b^* should be equally valid but would lead to a male-biased ESS allocation under *b* < 1 rather than a female bias as in Charnov's model [2]. If the sex allocation outcome depends on an arbitrary partition of the efficiency advantage of hermaphroditism between male and female functions, then a gamete-production interpretation of *x^b^* and 1 – *x* seems questionable.

## 5. Discussion

The Shaw–Mohler equation (2.1) was very meagre in its formulation but it contained (albeit implicitly) all the information needed to understand the processes that create selection on sex allocation. A translation from the inputs of males and females engaged in a mating arena (equation (2.1)) to the fitness outcomes from that arena (equation (3.2)) can provide an equivalent solution if fitness is derived in the same way. It is tempting to take equation (3.2) and suppose that when the mutant and all residents adopt allocation X* their relative male and female fitnesses will automatically be equal. But relative fitness does not arise in a vacuum; it has meaning only in terms of the total fitness from which it is derived, and if the fitness expressions do not satisfy the equality of total male and female fitness in a population, relative fitness derived from these expressions will lack biological legitimacy.

We are not compelled, of course, to accept the implicit panmixia of the Shaw–Mohler argument. Self-fertilization by a hermaphrodite or sexual differences in gamete dispersal are real phenomena that affect sex allocation. Power-function gain curves have a shape that can capture the consequences of these processes. For example, Fromhage & Kokko [27] report the fitness gain curves that result from sex-specific dispersal, but these gain curves are outcomes of an explicitly described mating process, not inputs to the model.

Fitness gain curves have great appeal because they represent common reproductive circumstances. Among angiosperms, for example, larger floral displays tend to be more but often only slightly more attractive to pollinators, so that average pollinator visitation per flower declines as inflorescence size increases [28–31]. In contrast, every additional flower produced by a wind-pollinated plant, not dependent on pollinator attention, might have essentially the same probability of pollination success as previous flowers [32]. Flowering patterns that prolong the temporal presentation of pollen might partially or wholly overcome the effects of inflorescence size on pollinator attraction [20]. All these ecological effects can be described by nonlinear functions, although possibly not power functions [2,4]. Gain curves may, then, be convenient descriptive summaries of real circumstances, including the effect of Bateman's principle.

At issue is not whether the shape of gain curves can describe real biology, but whether they are the source of frequency-dependent selection on sex allocation [3]. The Shaw–Mohler equation (2.1) implicitly but inexorably imposes frequency-dependent selection based on the numbers of male and female agents vying for mates, but Charnov's [2] gain-curve model conflated these numbers with fitness itself. Gain curves can describe the fitness outcomes from circumstances of reproductive ecology that create selection on sex allocation, but they are not underlying causes of sex allocation. It will therefore be necessary to revisit much theoretical and empirical work on sex allocation in hermaphrodites to ensure that causal efficacy is appropriately placed.

## Data Availability

This article has no additional data.
